# Parasitic Infections in African Humans and Non-Human Primates

**DOI:** 10.3390/pathogens9070561

**Published:** 2020-07-11

**Authors:** Hacène Medkour, Inestin Amona, Younes Laidoudi, Bernard Davoust, Idir Bitam, Anthony Levasseur, Jean Akiana, Georges Diatta, Liliana Pacheco, Slim Gorsane, Cheikh Sokhna, Raquel Adriana Hernandez-Aguilar, Amanda Barciela, Florence Fenollar, Didier Raoult, Oleg Mediannikov

**Affiliations:** 1 Aix Marseille Univ, IRD, AP-HM, MEPHI, 13385 Marseille, France; hacenevet1990@yahoo.fr (H.M.); younes.laidoudi@yahoo.com (Y.L.); bernard.davoust@gmail.com (B.D.); anthony.levasseur@univ-amu.fr (A.L.); didier.raoult@gmail.com (D.R.); 2 IHU Méditerranée Infection, 13385 Marseille, France; amoninestin@gmail.com (I.A.); georges.diatta@ird.fr (G.D.); cheikh.sokhna@ird.fr (C.S.); florence.fenollar@univ-amu.fr (F.F.); 3 PADESCA Laboratory, Veterinary Science Institute, University Constantine 1, El Khroub 25100, Algeria; 4 Aix-Marseille Univ, IRD, AP-HM, SSA, VITROME, 13385 Marseille, France; idirbitam@gmail.com; 5 Faculté des Sciences et Techniques, Université Marien Ngouabi, Brazzaville, Congo; 6 Superior School of Food Sciences and Food Industries, Algiers 16004, Algeria; 7 Laboratoire National de Santé Publique, Brazzaville, Congo; jakiana2000@yahoo.fr; 8 VITROME, IRD 257, Campus IRD/UCAD Hann, Dakar, Senegal; 9 Wara Conservation Project, Projet GALF-Guinée, 06110 Le Cannet, France; lilianaalbapacheco@gmail.com; 10 Direction Interarmées du Service de Santé des Armées des Forces Françaises Stationnées, Djibouti, East Africa; slim.gorsane@intradef.gouv.fr; 11 Jane Goodall Institute Spain and Senegal, Dindefelo Biological Station, Dindefelo, Kedougou, Senegal; r.a.hernandez-aguilar@ibv.uio.no (R.A.H.-A.); amanda.b@janegoodall.es (A.B.); 12 Department of Social Psychology and Quantitative Psychology, Faculty of Psychology, University of Barcelona, Passeig de la Vall d’Hebron 171, 08035 Barcelona, Spain

**Keywords:** nonhuman primates, humans, Nematoda, Mansonella, cross-species transmission, PCR

## Abstract

Different protozoa and metazoa have been detected in great apes, monkeys and humans with possible interspecies exchanges. Some are either nonpathogenic or their detrimental effects on the host are not yet known. Others lead to serious diseases that can even be fatal. Their survey remains of great importance for public health and animal conservation. Fecal samples from gorillas (*Gorilla gorilla*) and humans living in same area in the Republic of Congo, chimpanzees (*Pan troglodytes*) from Senegal and one other from the Republic of Congo, Guinea baboons (*Papio papio)* from Senegal, hamadryas baboons (*Papio hamadryas*) from Djibouti and Barbary macaques *(Macaca sylvanus*) from Algeria, were collected. DNA was extracted and screened using specific qPCR assays for the presence of a large number of helminths and protozoa. Positive samples were then amplified in standard PCRs and sequenced when possible. Overall, infection rate was 36.5% in all non-human primates (NHPs) and 31.6% in humans. Great apes were more often infected (63.6%) than monkeys (7.3%). At least twelve parasite species, including ten nematodes and two protozoa were discovered in NHPs and five species, including four nematodes and a protozoan in humans. The prevalences of *Giarida lamblia*, *Necator americanus, Enterobius vermicularis, Strongyloides stercoralis* were similar between gorillas and human community co-habiting the same forest ecosystem in the Republic of Congo. In addition, human specific *Mansonella perstans* (5.1%) and other *Mansonella* spp. (5.1%) detected in these gorillas suggest a possible cross-species exchange. Low prevalence (2%) of *Ascaris lumbricoides, Enterobius vermicularis, Strongyloides stercoralis* were observed in chimpanzees, as well as a high prevalence of *Abbreviata caucasica* (57.1%), which should be considered carefully as this parasite can affect other NHPs, animals and humans. The Barbary macaques were less infected (7.2%) and *Oesophagostomum muntiacum* was the main parasite detected (5.8%). Finally, we report the presence of *Pelodera* sp. and an environmental Nematoda DNAs in chimpanzee feces, *Nematoda* sp. and *Bodo* sp. in gorillas, as well as DNA of uncharacterized Nematoda in apes and humans, but with a relatively lower prevalence in humans. Prevalence of extraintestinal parasites remains underestimated since feces are not the suitable sampling methods. Using non-invasive sampling (feces) we provide important information on helminths and protozoa that can infect African NHPs and human communities living around them. Public health and animal conservation authorities need to be aware of these infections, as parasites detected in African NHPs could affect both human and other animals’ health.

## 1. Introduction

Parasitic infections cause a tremendous burden of disease in both the tropics and subtropics as well as in more temperate climates. There are three main groups of parasites that can cause disease in humans: protozoa, helminths and ectoparasites [1,2]. Parasitism is one of the most common disease entities threatening non-human primates (NHPs). Numerous protozoan and metazoan genera have been described, can infect different great apes and monkeys [3]. Some are nonpathogenic—or at least their detrimental effects on the host are not yet elucidated. However, a large number can lead to physiologic disorders, nutritional loss or produce lesions that result in severe damages, sometimes allowing sondary infections that may be fatal. Infection can be promoted by immunosuppression and various stressors [4].

Using the primate–parasite network, the role of different NHPs was evaluated for the probability of sharing infectious diseases with humans. Apes, as well as monkeys, such as baboons and macaques were shown to be infected with many parasites identified as emerging infectious diseases in humans [5]. Other studies have shown that parasites are frequently transmitted from wild or captive NHPs to humans in a shared habitat [6,7,8,9]. The protozoan *Giardia lamblia*, an enteric flagellate, induces diarrhea in monkeys and children [10]. *Entamoeba histolytica* has been described mainly in Old World NHPs including some apes (gibbons, orangutans and chimpanzees) as a cause of severe enteric disease [11]. It can infect humans as well, leading to dysentery. Cutaneous leishmaniasis agents were identified in NHPs, *Leishmania major* in wild gorillas [12] and *L. infantum* (visceral leishmaniasis) in New World NHPs [13]. Old World NHPs, such as chimpanzees and mangabeys can carry either *Schistosoma mansoni* or *S. hematobium* [14]. Parasites from the genus *Plasmodium* are among the best-studied parasites in African humans and NHPs, as they are responsible for malaria, the deadliest vector-borne disease [15]. The origin of the human malaria parasite *Plasmodium* was attributed to African apes. *Laverania* spp., found in various apes, belong to lineages in eastern chimpanzees as well as western lowland gorillas. They are nearly identical to *P. falciparum* and *P. vivax* [16,17]. Of note, *P. cynomolgi, P. siminovale* and *P. inui* are related to *P. vivax, P. ovale* and *P. malariae* in humans, respectively. Cross infection of *P. knowlesi* has also been documented in humans and NHPs [18,19,20,21]. Several other parasites that can be found in NHPs, such as *Babesia, Cryptosporidium*, *Amoeba, Toxoplasma, Trypanosoma*, Coccidia, nematodes and cestodes, possibly constitute a risk for humans [4,22,23].

For millennia, indigenous groups that depended on wildlife for their survival were exposed to the risk of NHP pathogens’ transmission. Inter species transmission of pathogens can occur through direct and indirect mechanisms. Direct mechanisms include hunting, fomites and wild meat consumption, keeping infected NHPs as pets or by eco-guardians, visitors or personal of primate center laboratories entering in direct contact with infected NHPs. Indirect mechanisms transmission of infectious pathogen stages through vectors such as blood sucking ticks, flies, fleas, sandflies, lice and tsetse flies transmitted pathogens such as *Trypanosoma* spp., *Bertiella* sp. tapeworm [3,24]. We still do not understand the dynamics of parasite interchange in detail but the increasing contact among species, may result in new ways of parasite interchange. By the way, in Uganda a study on how human impact the use of the ground and patterns of parasitism of *Pan troglodytes* suggest that the creation of trails and the increase of presence of humans in the forest results in increase in the frequency of use of the ground and higher parasite richness and intensity of infections [25]. The same results were found comparing groups of long-tailed macaques (*Macaca fascicularis*) living close to human modified environments and within the forests in Thailand [26]. No parasites present in humans were found in the macaques, but the macaques living in habitats modified by humans presented parasites (*Strongyloides fuelleborni* and probably *Haplorchis* sp.) that were not present in more isolated macaque groups. These last results are of importance, as the presence of humans and human related activities may modify the parasite community of non-human primates resulting in still unknown effects over their survival [27]. In this regard, Conly and Johnston (2008) suggest that this uncontrolled exposure of macaques to humans may have the potential for novel cross-species transmission of different parasites [28].

Finally, the high burden of zoonotic diseases continues to undermine the efforts and investments made for social and economic development. Therefore, developing strategies to ameliorate human and animal health through capacity building is pivotal for socioeconomic transformation and ranks top in developing country’s development agenda. Most zoonotic disease remains largely neglected in Africa and specially in sub Saharan Africa, probably because of distances existing between veterinary and medical professions. This lack of cooperation has in most cases left the burden of surveillance and control almost solely in the hands of veterinarian. Therefore, there is an urgent need for change of the status quo by adopting a holistic approach in controlling infectious diseases shared between humans and animals. As response to this need, in this study, we performed a survey of parasites (protozoa and helminths) using a fast typing technique by PCR in feces (noninvasive sampling method) of African NHPs. In an one health context and using the same approach, we examined a human population from the Republic of Congo living in the vicinity to gorillas in order to assess potential zoonotic transmission.

## 2. Results

**Figure 1:**
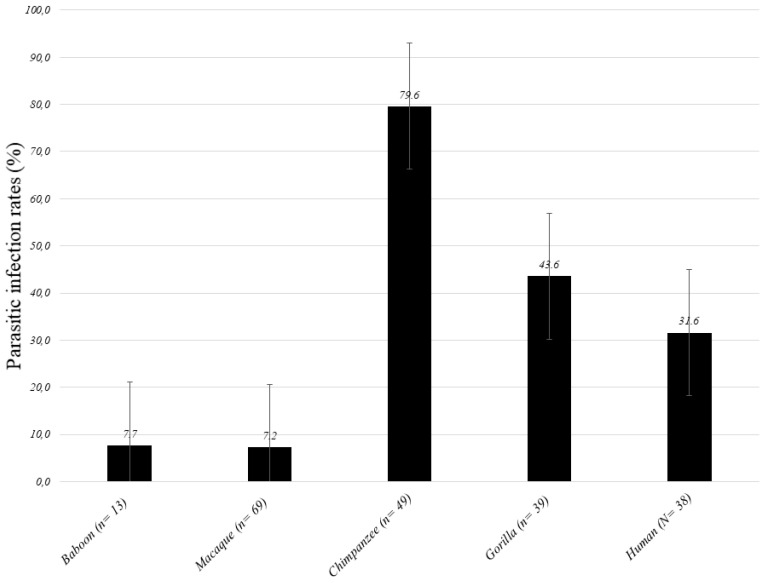
Results for parasite screening in feces from African humans and non-human primates (NHPs). Error bars represent the *SD*. Parasitic infection rates (%) mean the percentage of the presence of at least one infection.

In this study, the parasitic infection rate (i.e., presence of at least one parasitic infection) was 36.5% (62/170) in all NHPs. At least one infection was detected in 79.6% (39/49) of chimpanzees, 43.6% (17/39) of gorillas, 7.7% (1/13) of baboons and 7.2% (5/69) of macaques. Great apes were found to be more infested (63.6%) than monkeys (7.3%) (Z test, *p*-value < 0.0001) (Figure 1). In the Republic of Congo, infection rate was 43.6% (17/39) in gorillas and 31.6% (12/38) in the human community living close to them (Z test, *p*-value = 0.390), respectively.

At least one Nematoda infection was reported in 31.2% and 21.1% of NHPs and humans, respectively. Infection rates were (in NHPs and humans, respectively) 4.7% and 2.6% for *Filarioidea*, 2.9% and 13.2% for *Necator americanus*, 0.6% and 5.3% for *Ascaris lumbricoides*, 2.4% and 2.6% for *Strongyloides stercoralis. Abbreviata caucasica* von Linstow (Physalopteridae: Spirurida) (Syn *Physaloptera caucasica*) was detected only in Senegalese wild chimpanzees (57.1%), as reported in our previous study [29]. *Enterobius vermicularis* nematode was identified only in one gorilla and one chimpanzee, thus in 1.2% of NHPs.

DNA of *Mansonella* spp. has been detected in four gorilla samples from the Republic of Congo. Among them, two were identified as *M. perstans*. In this study, DNA of uncharacterized nematodes was detected in 12 (7.1%) NHPs, including 10 chimpanzees (20.4%), one gorilla (2.6%), one Guinea baboon (7.7%) and in one human (Table 1). Except for filarioid infections (Z test, *p*-value = 0.028), no significant difference was observed for the presence of Nematoda in gorillas and humans in the Republic of Congo.

**Figure 2:**
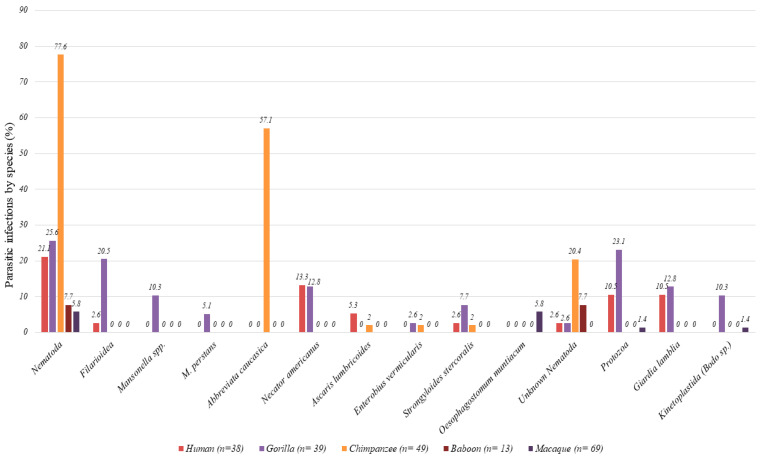
Parasitic infection by species of NHPs and humans.

Protozoan infections were also present in 5.9% of NHPs, including almost all in gorillas (23%) and in 10.5% of humans (Z test, *p*-value: 0.014). *Giardia lamblia* was detected in 12.5% of gorillas and in 10.5% of humans, while Kinetoplastida were detected in 10.3% of gorillas and in one macaque 1.4% (Figure 2, Table 1).

Except for *A. caucasica* (Z test, *p*-value < 0.0001*), no significant differences in infection rates between humans and NHPs were observed. The other pathogens searched in this study were not detected.

**Figure 3:**
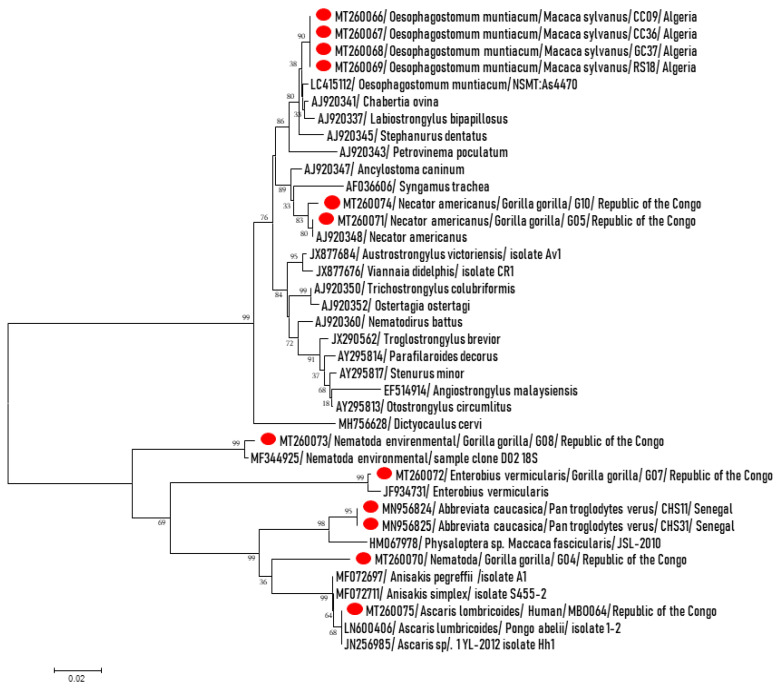
Phylogenetic tree representing *Nematoda* spp. detected in this study from African primates and humans.

Furthermore, after PCR/sequencing of qPCR positive samples for the partial Nematoda 18S gene (Figure 3), in addition to confirming the qPCR results, DNA from *Oesophagostomum* sp. was identified in four (4.2%) Barbary macaques. These sequences of ~1100 bps from 18S rRNA, were almost identical to each other and exhibited > 99.3 identity with *O. muntiacum* NSMT (LC415112) detected in large intestine of Reeves’s muntjac (*Muntiacus reevesi*) from Izu-Oshima Island, Tokyo, Japan. Using PCR primers for 28S pan-helminths, *O. muntiacum* was confirmed by amplifying ~500 bps of the 28S gene from all positive samples by pan-Nematoda 18S PCR. These sequences were almost similar and allowed 99.7% similarity with *O. muntiacum* NSMT (LC415112). Almost all of the chimpanzee positive samples by qPCR for *A. caucasica* were amplified and ~1200 bps fragments were obtained. They were almost identical and were identified as *A. caucasica*. Simultaneously, the COI genes were amplified, they were almost identical to each other and were identified as *A. caucasica* as described previously [29].

The evolutionary history based on 18S rRNA partial gene was inferred using the neighbor-joining method. The optimal tree with the sum of branch length = 0.71539928 is shown. The confidence probability (multiplied by 100) that the inside length of the branches is greater than 0, as estimated by the bootstrap test (1000 replicates), is shown next to the branches. The tree is drawn to scale, with branch lengths in the same units as those of the evolutionary distances used to infer the phylogenetic tree. Sequences obtained in this study are highlighted by red points in the beginning, Nematoda species, NHP species sample ID and country. They are compared to the related Nematoda sequences from GenBank database. The evolutionary distances were computed using the Tamura–Nei method and are in the units of the number of base substitutions per site. The analysis involved 38 nucleotide sequences. All positions containing gaps and missing data were eliminated. Evolutionary analyses were conducted in MEGA7.

Three other sequences of ~400 bps of 18S gene from gorillas were 100% identical to *N. americanus* (AJ920348), all of these samples were also positive by specific qPCR for *N. americanus*; (E) *vermicularis* qPCR-positive samples were confirmed by amplifying a 400-bps sequence showing 99.2% homology with *E. vermicularis* (JF934731). In addition, small good quality sequences of approximately 200 bps were obtained from positive gorilla *M. perstans* qPCR samples. They were similar and exhibited 98.5% similarity with *M. perstans* isolate PlasNew649 (MN821068) found in the blood of a Gabonese man. Finally, a DNA sequence was obtained from one of the two positive human *A. lumbricoides* qPCR samples, it displayed 99.8% similarity to *A. lumbricoides* (LN600406).

We detected a *Rhabditidae* nematode on a chimpanzee. The 320-bps fragment of 28S partial gene amplified was 82% similar to saprophytic nematode *Pelodera cylindrica* (EU195994). A fragment of the 18S gene, approximately 400 bp in length, was obtained from a gorilla that did not show any nematode-like identity. The closest was only 94% of similarity with *Anisakis simplex* (MF072711) and *A. pegreffii* (MF072697). An environmental nematode showing 99.5% identity with Plectidae sp. MK-2017 (LC275886) was detected in a gorilla, we do not exclude environmental contamination since the fecal samples were taken from the soil.

All positive samples in qPCR pan-Kinetoplastida were identified by sequencing of 18S and 28S partial genes as *Bodo* spp., a free living nonpathogenic Kinetoplastida.

## 3. Discussion

Overall, when parasites (helminths and protozoa) were searched in the feces of NHPs and a nearby indigenous human population using molecular tools, a wide range of parasites belonging to the Nematoda and Kinetoplastida classes were detected. Parasitism rate was almost similar in both humans and gorillas co-habiting the same ecosystem forest in the Republic of Congo and nematode infections were the most predominant. The prevalence was 8.7-fold higher in great apes than monkeys. In addition, sub-Saharan African primates were 7.3-fold more infected than macaques from North Africa. The raison of this difference is not clear, and since infectious diseases are modulated by ecosystems, we think that primates in sub-Saharan Africa are more exposed to these diseases because this region is characterized by the greatest infectious disease burden as well as by the weakest public health infrastructure in the world [30]. The method used here (PCR) was more sensitive than microscopy for parasite detection in patients without gastrointestinal symptoms [31]. By contrast, we are aware of the low sensitivity of specific PCR on feces for the detection of extraintestinal parasites, such *Plasmodium*, Filaria and Kinetoplastida etc., particularly parasites of blood. Consequently, prevalence of extraintestinal parasites is underestimated in this study.

Our results are complementary to a study conducted in Gabon in the Lopé Reserve, which revealed the presence of protozoa, trematodes and nematodes in 84% of gorilla samples and 56% of chimpanzee samples [32]. Another study, conducted in the Dzanga-Ndoki National Park in the Central African Republic, described both helminths and protozoa in fecal samples from great apes (gorillas, chimpanzees) and human populations (Ba’Aka, indigenous Bantu and western researchers) [33].

*Giardia lamblia* (syn *G. intestinalis, G. duodenalis*), a parasite of the protozoan group, has been found in feces of great apes (gorillas), as well as in humans living around them in this study, suggesting possible interactions. NHPs can be asymptomatic carriers and hence be a source to human infections, with infection via direct contact [34]. Evidence of possible fecal–oral transmission of *Giardia* between apes and their attendants was reported from the Kansas City Zoo. Clinical signs in Kansas City outbreak consisted of diarrhea and vomiting in both non-human and human patients [4]. Although work continues on speciation and host-specificity in *Giardia*, studies have shown that at least some species of *Giardia* are transmissible from animals to humans and vice versa, thus making it potentially both a zoonotic and an anthropozoonotic infection [35,36]. In the studies conducted by Nolan et al. in Uganda and Drakulovski et al. in Cameroon, *G. lamblia* was absent in mountain gorilla and chimpanzee samples, while 9.1% of humans were infected with this protozoan [37,38]. *Giardia*, once considered a harmless parasite for humans and animals, is now recognized as a pathogenic agent [4]. *G. lamblia* occurs worldwide and is a common inhabitant of the small intestine of humans, rhesus monkeys, cynomolgus monkeys, chimpanzees, gorillas and other NHPs [39,40,41]. We also detected in feces of NHPs *Bodo* spp., a free-living nonpathogenic flagellate. Other pathogenic protozoa reported in feces of non-human and human primates, such as *Plasmodium*, *Leishmania, Trypanosoma, Cryptosporidium, Toxoplasma* and *Babesia* [4,22] have not been detected in the present study. Except for *Cryptosporidium*, the other parasites have an extraintestinal localization, so do not found them in stools is not surprising and samples as blood will be more suitable for their surveillance.

As for helminths, at least ten *Nematoda* species were detected in NHPs versus four species in humans in this study. In gorillas, filariid infections (including *Mansonella* spp.) were the most prevalent, followed by *N. americanus* and *S. stercoralis* which were detected in human co-habited gorillas.

A wide range of filariid nematodes has been reported in great apes and monkeys [4]. Interestingly, we detected DNA of *M. perstans* in two gorilla stools and *Mansonella* spp. in two others. Several non-perstans *Mansonella* were reported in apes from Congo and neighboring countries. This includes: *Mansonella* (*Esslingeria*) (Chabaud & Bain, 1976), *M. (E.) leopoldi* from *Gorilla gorilla* in the Republic of the Congo [42] and Gabon [43], *M. (E.) lopeensis* from *Gorilla gorilla* in Gabon [43], *M. (E.) vanhoofi* from *Pan paniscus* in DR Congo [44,45] and from *Gorilla gorilla* in the Republic of Congo [42]; (M) *(E.) streptocerca* and *M. (E.) rodhaini* are two filariid parasites reported from chimpanzees and gorillas [46,47,48]. Chimpanzees in Central Africa are the reservoir of *M. rodhaini*, which was identified in skin biopsy samples from several villagers in Gabon [49]. *M. (E.) vanhoofi*, a filariid parasite of the chimpanzee, inhabits the mesenteries and the connective tissue adjacent to the gallbladder, bile duct, liver, pancreas and kidney and the loose connective tissues and lymphatics surrounding the hepatic blood vessels [4,46]. Three *Mansonella* species often infect humans: *M. perstans, M. ozzardi* and *M. streptocerca*. Whereas *M. perstans* has never been reported to infect NHPs, except a report in *Gorilla gorilla* and *Pan troglodytes* in Cameroon (Reichenow 1917) [50]. *M. streptocerca* has been found in primates [48]. Based on our findings, it will be very important to study *Mansonella* infections in great apes to achieve a better understanding of this genus.

Several other filariids have been reported from the great apes, including *Dirofilaria immitis* from the heart of an orangutan [51] and in the abdominal cavity of another orangutan [52] and *Loa loa* and *Onchocerca volvulus* from chimpanzees and gorillas [4,42,53], but none of them were detected in this study.

In our study, the identified infection rate for *S. stercoralis* was lower compared with that reported by Lilly et al. (2002) (82–94% in NHPs and 30–93% in humans) [33]. It was also lower than the prevalence 74.3% and 100% observed in two chimpanzee communities in Gombe National Park, Tanzania [53] and one in Kibale National Park, Uganda [54], respectively. In contrast, the prevalence of *Strongyloides* in gorillas in our study was higher than the prevalence (1.4%) in gorilla populations from Rwanda [22] and almost similar to the prevalence (10.9%) in chimpanzees from Rubondo National Park, Tanzania [55]. These results contrast with those of Hasegawa et al. who reported the presence of *S. stercoralis* in fecal samples from local human populations and the absence of this parasite in wild western lowland gorillas (*Gorilla gorilla*) and a central chimpanzee (*Pan troglodytes troglodytes*) living in the Dzanga-Sangha Protected Areas (DSPA), Central African Republic and in eastern chimpanzees (*Pan troglodytes schweinfurthii*) living in degraded forest fragments on agricultural land in Bulindi, Uganda [37,56]. Conversely, a study carried out in 2017 in Thailand revealed the presence of *S. stercoralis* in human communities in contact with long-tailed macaques with a prevalence of 8.92% [57].

In the case of *N. americanus*, the prevalence observed in this study in great apes and humans is lower than the rates of 86% in wild lowland gorillas in Cameroon [58] and 44% in chimpanzees from Uganda [37]. Our study concords with the study of Hasegawa et al. conducted in Central African Republic. It has been shown that *Necator* hookworms are shared by humans and great apes co-habiting the same tropical forest ecosystems [59]. Another nematode detected in both apes (Chimpanzee from Rep. of Congo) and humans in our study was *A. lumbricoides* with low infection rates. These rates are lower than the rates of *Ascaroides* observed by Lilly et al. (2002) in apes (14–88%) and humans (0–15%). *E. vermicularis* was detected with a low prevalence only in apes (2.3%). *E. vermicularis* and other *Enterobius* species found in Old World monkeys and great apes as well as humans, *E. anthropopitheci* in the chimpanzees and in several species of prosimian primates. These parasites are considered cosmopolitan in their geographical distribution [4]. Naturally infected NHPs could be sources of infection for humans. In addition, captive primates can acquire *E. vermicularis* infection from humans and then can act as reservoirs to reinfect humans [39,60].

*A. caucasica* has been detected with high prevalence (57%) only in wild Senegalese wild chimpanzees as reported in Laidoudi et al. (submitted). Physalopteriasis, is a disease caused by members of the genus *Physaloptera*. Nine species of physalopterids have been reported to occur in the upper gastrointestinal tract of NHPs [51]. *A. caucasica* has been found in the esophagus, stomach and small intestine of the rhesus macaques, baboons and orangutans [39,42,61,62,63]. This parasite can be transmitted to humans, but complete life-cycle is not identified until now. It has been reported from humans in Brazil, Colombia, Congo Republic (Zaire), India, Indonesia, Israel, Namibia, Panama, Zambia and Zimbabwe [4]. In one case, in an Indonesian woman, adult worms were recovered from the bile duct, where they had caused biliary pain, jaundice and fever [4].

In addition, *O. muntiacum* was detected in 4.3% of North African macaques in the present study. Oesophagostomiasis is caused by infection of nematodes from the genus *Oesophagostomum*, the nodular worm. Eggs are shed in the feces of the definitive host and may be indistinguishable from the eggs of *Necator* and *Ancylostoma*. Eggs hatch into rhabditiform (L1) larvae in the environment, given appropriate temperature and level of humidity. In the environment, the larvae will undergo two molts and become infective filariform (L3) larvae. Worms can go from eggs to L3 larvae in a matter of a few days, given appropriate environmental conditions. Definitive hosts become infected after ingesting infective L3 larvae. After ingestion, L3 larvae burrow into the submucosa of the large or small intestine and induce cysts. Within these cysts, the larvae molt and become L4 larvae. These L4 larvae migrate back to the lumen of the large intestine, where they molt into adults. Eggs appear in the feces of the final host approximately one month after ingestion of infectious L3 larvae (https://www.cdc.gov/dpdx/oesophagostomiasis/index.html). These parasites are considered the most common nematodes found in Old World monkeys and great apes, which constitute the definitive hosts. They have been described in baboons, mangabeys, guenons, macaques, chimpanzees and gorillas [4,64,65]. They are rare in New World monkeys [4,66,67]. *Oesophagostomum* spp. parasite normally ruminants, pigs and monkeys and occasionally humans [68]. It has been shown that multiple cryptic forms of *Oesophagostomum* circulate in populations of primates in western Uganda, and that parasitic clades differ in terms of host range and potential transmission between species [69].

Finally, using the high-sensitive PCR systems, we detected—among other things—three unidentifiable genotypes. Of them, two may belong to the environmental nematodes such *Pelodera* sp. and an environmental *Nematoda* sp. in chimpanzee feces. This may be an environmental contamination since samples were collected on the ground. One *Nematoda* sp. we detected in gorillas remains difficult to interpret. On one hand, the gene does not correspond to any known species deposited in the GenBank, on the other hand, it is grouped among the other pathogens of the phylogenetic tree (Figure 2), it can thus belong to a pathogenic species but not described or not characterized. Further, we detected DNA of uncharacterized Nematoda in apes and humans, but with a relatively lower prevalence in humans. Characterization of these parasites by standard PCR-sequencing was not possible due to co-infections with more nematodes in the same sample and/or small amounts of DNA since most of the collected NHPs samples are degraded. In addition, DNA sequences of wild parasite range are not available, which makes the molecular characterization difficult. This requires further molecular studies, parasite characterization and database enrichments.

The present study remains interesting on the knowledge of parasites of NHPs and humans in Africa. One of its strengths that we performed a large PCR screening on samples from different countries using a non-invasive sampling method. In addition, different great ape and monkey species were involved, as well as humans in contact with great apes to look at the zoonotic risk. Nevertheless, it has some limitations such as: the low number of samples for some species (baboons and green monkeys), underestimated prevalence of extraintestinal parasites (*Plasmodium*, Filaria, Kinetoplastida etc.), since feces are not the preferred sampling method. These limits require further investigation in future studies using other samples such blood.

## 4. Material and Methods

### 4.1. Animals and Study Area

Feces from humans and NHPs were collected (Table 2). The sampling was non-invasive and did not harm the wild fauna. For NHPs, fecal samples were collected at sleeping sites, feeding sites and places where the primates had been observed. Gorilla and macaque samples, ten from chimpanzees and samples from hamadryas baboons were degraded stored in absolute alcohol. Thirty-eight chimpanzee and all human stool samples were collected in the fresh state. All degraded samples were stored in absolute alcohol, and fresh samples and human samples were first stored at −20 °C before being sent from the Republic of Congo to France for analysis.

All humans that participated in the study were apparently healthy, the health status of animals is unknown. All samples collected were brought to the IHU Méditerranée Infection laboratory, Marseille, France, where they were stored at −20 °C or −80 °C until further analysis.

### 4.2. Ethic Statement

Study authorizations were obtained from the Direction National of Parks (DNP) in the Senegalese Ministry of the Environment (DNP, No. 1302, Oct 16, 2015) for Senegalese primates, the Ministry of Health (No 208/MSP/CAB.15 of 20 August 2015) and the Forest Economy and Sustainable Development (No 94/MEFDD/CAB/DGACFAP-DTS of 24 August 2015) of the Rep. of Congo for humans and gorillas respectively, the Center for Studies and Research of Djibouti for baboons (*Papio hamadryas*) and the management of the Chréa National Park (CNP) in Blida Province, Algeria, for Barbary macaques. No experimentation was conducted on NHPs, as fecal samples were collected from the soil. Human samples were taken after obtaining verbal consents of participants due to their low literacy.

### 4.3. DNA Extraction

DNA extraction was performed using the EZ1^®^DNA tissue kit (Qiagen, Hiden, Germany) on BIOROBOT EZ1 (Qiagen, Hiden, Germany), according to the manufacturer’s instructions. Initially, we mixed in tubes about 200 mg of stool with 360 µL of G2 lysis buffer (Qiagen, Hiden, Germany). This was mechanically lysed with tungsten beads (Qiagen, Hiden, Germany) using FastPrep-24TM 5G Grinder for 40 s. After 10 min of incubation at 100 °C to allow for complete lysis, tubes were centrifuged at 10,000× *g* for 1 min. Subsequently, 200 μL of supernatant was enzymatically digested using 20 μL of proteinase K (20 mg/mL, Qiagen) and incubated overnight at 56 °C. DNA was extracted from 200 µL of sample, eluted in 200 µL volume, then aliquoted in individual tubes of: pure extracted DNA, diluted to 1:10 and finally DNA diluted to 1:100.

To control the extraction quality and the absence of PCR inhibitors, universal eubacterial qPCR targeting the 16S rRNA bacterial genes, named “qPCR all bacteria” [70], was performed on pure DNA, dilutions to1:10 and to 1:100. By comparison of Ct values obtained for pure and diluted DNAs, the dilution to1:10 were chosen for parasite screening. DNA tubes were stored at −20 °C until use.

### 4.4. Molecular Screening for Parasites by Real-Time PCR Assays (qPCR)

DNA dilutions to 1:10 were screened for parasites in order to evade the nonspecific PCR inhibition. We have used single target real-time PCR assays specific for each pathogen. Eight qPCR assays targeting a large number of parasite members (class, order or genus) and seventeen different species—specific primers and Taqman probes (hydrolysis probes), all known for their specificity and sensitivity, were used in multiparallel assays, as shown in Table 3.

The qPCR amplifications were performed in a CFX96 Real-time system (BioRad Laboratories, Foster City, CA, USA). Reactions were performed in a volume of 20 µL, containing 5 µL of DNA template, 10 µL of Master Mix Roche (Eurogentec, Seraing, Belgium), 0.5 µL each primer per reaction at the concentration of 20 µM, 0.5 µL UDG and 0.5 µL of each probe at the concentration of 5 µM. The TaqMan cycling conditions included two hold steps at 50 °C for 2 min, followed by 95 °C for 15 min, and 40 cycles of two steps each (95 °C for 30 s and 60 °C for 30 s). Each PCR plate contains 96-wells. Known DNAs or plasmids were used as positive controls and master mixtures as a negative control in each reaction.

### 4.5. Genetic Amplification by Standard PCR, Sequencing and Phylogeny

In addition to species-specific qPCR screening, we performed screening by broad-range qPCRs for pan-Nematoda and for pan-Kinetoplastida parasites, followed by PCR/ sequencing. Positive samples in pan-Nematoda qPCR were subjected to standard PCRs targeting 18S rRNA gene of Nematoda and the 28S rRNA gene of helminths. Positive ones for Kinetoplastida qPCR were amplified using primer pairs for 18S and 28S genes of Kinetoplastida (Table 3). Amplifications were carried out in a total volume of 50 µL, consisting of 25 µL of AmpliTaq Gold master mix, 18 µL of ultra-purified water DNAse-RNAse free, 1 µL of primers (20 µM of concentration) and 5 µL of DNA template. The thermal cycling conditions amplifications were as follows: incubation step for 15 min at 95 °C, 40 cycles of: one minute at 95 °C, 30 s for at the annealing temperature (Table 3), an elongation step at 72 °C. Finally, an extension step for five minutes at 72 °C. This was performed in a Peltier PTC-200 model thermal cycler (MJ Research, Inc., Watertown, MA, USA) and visualized on 2% agarose gel. In a second time, amplicons were purified using NucleoFast 96 PCR plates (Macherey–Nagel EURL, Hoerdt, France) as per the manufacturer’s instructions and sequenced using the Big Dye Terminator Cycle Sequencing Kit (PerkinElmer Applied Biosystems, Foster City, CA, USA) with an ABI automated sequencer (Applied Biosystems). Generated electropherograms were assembled and edited using ChromasPro software (ChromasPro 1.7, Technelysium Pty Ltd., Tewantin, Australia) and compared with those available in the GenBank database by NCBI BLAST (https://blast.ncbi.nlm.nih.gov/Blast.cgi). The fragments obtained were compared with each other and with related fragments available in the GenBank database. The phylogenetic analyses were inferred using neighbor joining methods and tree reconstructions were performed using MEGA software version 7 (https://www.megasoftware.net/). Bootstrap analyses were conducted using 1000 replicates.

## 5. Conclusions

This study provides data on different helminths and protozoa that infect NHPs in Africa and human communities living around them. Parasites known to infect both humans and NHPs have been detected in humans and gorillas living in the same tropical forest ecosystem, suggesting possible interactions. In addition, human specific parasites, such as *Mansonella perstans*, causative agent of one of the major human neglected tropical diseases, was detected in gorillas, suggesting an exchange between humans and NHPs and other investigations are required at this stage for better understanding these findings. However, prevalence of extraintestinal parasites remain underestimated since feces are not the preferable sampling method and samples such as blood can give more information. In addition, public health and animal conservation authorities need to be aware of these infections, as the parasites observed in African NHPs could affect both human and animal health.

## Figures and Tables

**Table 1:** Parasites detected in the present study and their prevalence, *n* (%).

*Species* *(n)*	Gorilla (*n* = 39)	Chimpanzee (*n* = 49)	Baboon (*n* = 13)	Macaque (*n* = 69)	NHPs (N = 170)	Human (N = 38)	*Difference:* *p*-Value
Parasitic infections	17(43.6)	39 (79.6)	1 (7.7)	5 (7.2)	62 (36.5)	12 (31.6)	*0.390*
Nematoda	10 (25.6)	38 (77.6)	1 (7.7)	4 (5.8)	53 (31.2)	8 (21.1)	*0.836*
*Filarioidea*	8 (20.5)	0	0	0	8 (4.7)	1 (2.6)	*0.028*
*Mansonella* spp.	4 (10.3)	0	0	0	4 (2.4)	0	*0.115*
*M. perstans*	2 (5.1)	0	0	0	2 (1.2)	0	*0.474*
*Abbreviata caucasica*	0	28 (57.1)	0	0	28 (16.5)	0	*<0.0001*
*Necator americanus*	5 (12,8)	0	0	0	5 (2.9)	5 (13.2)	*1.000*
*Ascaris lumbricoides*	0	1 (2)	0	0	1 (0.6)	2 (5.3)	*0.462*
*Enterobius vermicularis*	1 (2.6)	1 (2)	0	0	2 (1.2)	0	*1.000*
*Strongyloides stercoralis*	3 (7.7)	1 (2)	0	0	4 (2.4)	1 (2.6)	*0.622*
*Oesophagostomum muntiacum*	0	0	0	4 (5.8)	4 (2.4)	0	*1.000*
*Unknown* Nematoda	1 (2.6)	10 (20.4)	1 (7.7)	0	12 (7.1)	1 (2.6)	*1.000*
***Protozoa***	9 (23.1)	0	0	1 (1.4)	10 (5.9)	4 (10.5)	*0.014*
*Giardia lamblia*	5 (12.8)	0	0	0	5 (2.9)	4 (10.5)	*1000*
Kinetoplastida *(Bodo sp.)*	4 (10.3)	0	0	1 (1.4)	5 (2.9)	0	*0.115*

*p*-value: between humans and NHPs from the Republic of Congo living in the same area.

**Table 2:** Study subjects and study sites.

Species	Country	Region	Coordinates	Number (date)
**Non-human primates**				160 (2015–2018)
**Chimpanzee** **(*Pan troglodytes*)**	Senegal	Kédougou	12°22′57.1404″N 12°17′16.7172″W	3 (2016)
			12°22′53.1732″N 12°17′26.7936″W	7 (2016)
			12°22′47.7084″N 12°17′48.588″W	38 (2016)
	Rep. Congo	Odzala-Kokoua NP	1.3206°”N 14.8455°”E	1 (2017)
**Gorillas** **(*Gorilla gorilla*)**	Rep. Congo	Lésio-Louna NP	2°58′33.1”S 15°28′33.4”E	16 (2015), 12 (2017)
		Odzala-Kokoua NP	1.3206°”N, 14.8455°”E	10 (2017)
		Nouabale-Ndoki NP	2.5857°”N, 16.6291°”E	1 (2017)
**Baboons** **(*Papio papio*)**	Senegal	Niokolo-Koba NP	13°04′28.6”N 12°43′18.2”W	7 (2015)
**Baboons** **(*Papio hamadryas*)**	Djibouti	Oueah	11°29′56.1”N 42°51′14.8”E	6 (2017)
**Barbary macaques (*Macaca sylvanus)***	Algeria	Chréa NP	36°23′42.9”N 2°45′53.6”E	30 (2018)
		Cap Carbon	36°46′31.6”N 5°06′11.2”E	39 (2018)
Humans	Rep. Congo	Mbomo village	1.3206°”N, 14.8455°”E	35 (2017)
		Lésio-Louna (Eco-guards)	2°58′33.1”S 15°28′33.4”E	3 (2017)

NP: national park

**Table 3:** Primer and probe sequences used in this study.

Parasite	Target Gene	Primer Name	Sequence (5′-3′)	Source
Kinetoplastida	28s	F. 24a.5198	AGTATTGAGCCAAAGAAGG	[71]
		R. 24a.5412	TTGTCACGACTTCAGGTTCTAT	
		P. 24a.5345	FAM-TAGGAAGACCGATAGCGAACAAGTAG-TAMRA	
*Leishmania* spp.	18s	F	GGTTTAGTGCGTCCGGTG	[72]
		R	ACGCCCCAGTACGTTCTCC	
		P	FAM-CGGCCGTAACGCCTTTTCAACTCA-TAMRA	
*Trypanosoma* spp.	5.8s	F. 5.8 S Tryp 3874	CAACGTGTCGCGATGGATGA	
		R. 5.8 S Tryp 3935	ATTCTGCAATTGATACCACTTATC	
		S. 5.8 S Tryp 3911	FAM-GTTGAAGAACGCAGCAAAGGCGAT-TAMRA	
Piroplasmida	5.8s	5.8S-F5	TCGCAGRAGTCTKCAAGTC	[73]
		5.8S-R	AYYKTYAGCGRTGGATGTC	
		5.8S-S	FAM-TTYGCTGCGTCCTTCATCGTTGT-MGB	
*Cyclospora cayetanensis*	18s	Cyclo250F	TAGTAACCGAACGGATCGCATT	[74]
		Cyclo350R	AATGCCACGTAGGCCAATA	
		Cyclo281T	FAM-CCGGCGATAGATCATTCAAGTTTCTGACC-TAMRA	
*Plasmodium* spp.	Cox	Plasmo_cox_15_F	AGGAACTCGACTGGCCTACA	[75]
		Plasmo_cox_16_R	CCAGCGACAGCGGTTATACT	
		Plasmo-cox_P	FAM-CGAACGCTTTTAACGCCTGACATGG-TAMRA	
*Toxoplasma gondii*	ITS1	Tgon_ITS1_F	GATTTGCATTCAAGAAGCGTGATAGTA	[76]
		Tgon_ITS1_R	AGTTTAGGAAGCAATCTGAAAGCACATC	
		Tgon_ITS1_P	FAM-CTGCGCTGCTTCCAATATTGG-TAMRA	
*Cryptosporidium parvum; C. hominis*	hsp70 gene	1PSF	AACTTTAGCTCCAGTTGAGAAAGTACTC	[77]
		1PSR	AACTTTAGCTCCAGTTGAGAAAGTACTC	
		Crypt P	FAM-AATACGTGTAGAACCACCAACCAATACAACATC-TAMRA	
*Giardia lamblia (intestinalis or duodenalis)*	18s	Giardia-80F	GACGGCTCAGGACAACGGTT	[78]
		Giardia-127R	TTGCCAGCGGTGTCCG	
		Giardia-105T	FAM-CCCGCGGCGGTCCCTGCTAG-TAMRA	
*Entamoeba histolytica*	18s	Ehf	AACAGTAATAGTTTCTTTGGTTAGTAAAA	[79]
		Ehr	CTTAGAATGTCATTTCTCAATTCAT	
		Ehp	FAM-ATTAGTACAAAATGGCCAATTCATTCA-TAMRA	
Nematoda	5s	qNem.5S.1f	ACCACGTTGAAAGCACGMC	[29]
		qNem.5S.110r	TGTCTACAACACCTSGRATTCC	
		qNem.5S.38p	FAM-AGTTAAGCAACGTTGGGCC-TAMRA	
*Filariae*	28S	qFil-28S-F	TTGTTTGAGATTGCAGCCCA	[80]
		qFil-28S-R	GTTTCCATCTCAGCGGTTTC	
		qFil-28S-S	FAM-CAAGTACCGTGAGGGAAAGT-TAMRA	
*Mansonella* spp.	ITS1	Forward	CCTGCGGAAGGATCATTAAC	[81]
		Reverse	ATCGACGGTTTAGGCGATAA	
		Probe	FAM-CGGTGATATTCGTTGGTGTCT-TAMRA	
*Mansonella perstans*	ITS1	Forward	AGGATCATTAACGAGCTTCC	
		Reverse	CGAATATCACCGTTAATTCAGT	
		Probe	FAM-TTCACTTTTATTTAGCAACATGCA-TAMRA	
*Loa loa*	LL20 15-kDa ladder antigen	15r3-5	CGAAAAATTATAGGGGGAAAC	[82]
		15r3-6	TCGTAGACCAAACTGCGAAC	
		15r3-P	FAM-TCAAGAGCCGATATACTGAAAGCTATC-TAMRA	
*Abbreviata caucasica*	12s	Phy.12S.f.204	GAATTGGATTAGTACCCAAGTAAGTG	[29]
		Phy.12S.r.305	TGTTCCAAAAATCTTTCTAAGATCAG	
		Phy.12S.242p	VIC-GCGGGAGTAAAGTTAAGTTTAAACC-TAMRA	
		Cyclo350R	AATGCCACGTAGGCCAATA	
		Cyclo281T	FAM-CCGGCGATAGATCATTCAAGTTTCTGACC-TAMRA	
*Necator americanus*	ITS2	Na58F	CTGTTTGTCGAACGGTACTTGC	[83]
		Na158R	ATAACAGCGTGCACATGTTGC	
		Na81T	FAM-CTGTACTACGCATTGTATAC-MGB	
*Ancylostoma duodenale*	ITS2	Ad125F	GAATGACAGCAAACTCGTTGTTG	
		Ad195R	ATACTAGCCACTGCCGAAACGT	
		Ad155-XS	FAM-ATCGTTTACCGACTTTAG-MGB	
*Schistosoma mansoni*	Tandem repeat units M61098	SRA1	CCACGCTCTCGCAAATAATCT	[84]
		SRS2	CAACCGTTCTATGAAAATCGTTGT	
		SRP	FAM-TCCGAAACCACTGGACGGATTTTTATGAT-TAMRA	
*Taenia solium*	ITS	Tsol_145F	ATGGATCAATCTGGGTGGAGTT	[85]
		Tsol_230R	ATCGCAGGGTAAGAAAAGAAGGT	
		Tsol_169Tq	FAM-TGGTACTGCTGTGGCGGCGG-TAMRA	
*Taenia saginata*	ITS	Tsag_F529	GCGTCGTCTTTGCGTTACAC	
		Tsag_R607	TGACACAACCGCGCTCTG	
		Tsag_581Tq	FAM-CCACAGCACCAGCGACAGCAGCAA-TAMRA	
*Ascaris lumbricoides*	ITS1	Alum96F	GTAATAGCAGTCGGCGGTTTCTT	
		Alum183R	GCCCAACATGCCACCTATTC	[86]
		Alum124T	FAM-TTGGCGGACAATTGCATGCGAT-TAMRA	
*Trichuris trichiura*	18s	TrichF	TTGAAACGACTTGCTCATCAACTT	[87]
		TrichR	CTGATTCTCCGTTAACCGTTGTC	
		TrichP	FAM-CGATGGTACGCTACGTGCTTACCATGG-TAMRA	
*Strongyloides stercoralis*	18s	Stro-1530F	GAATTCCAAGTAAACGTAAGTCATTAGC	[88]
		Stro-1630R	TGCCTCTGGATATTGCTCAGTTC	
		Stro-1586T	FAM-ACACACCGGCCGTCGCTGC-TAMRA	
*Enterobius vermicularis*	5s	EnterF	TTTCCAAGCCACAGACTCAC	
		EnterR	ATTGCTCGTTTGCCGATTAT	[31]
		EnterP	TCATGTCTGAGCCGGAACGAGA	
Nematoda	18s	Fwd.18S.631	TCGTCATTGCTGCGGTTAAA	[89]
		Rwd.18S.1825r	GGTTCAAGCCACTGCGATTAA	
Helminths	28s	Hspec.28S. 5748f	GGTAAGGGAAGTCGGCAAAT	This study
		Hspec.28S.6394r	TAGGGACAGTGGGAATCTCG	
*Abbreviata caucasica*	COI	F.Abbrev.COI.51f	TGATCAGGGTTGGGAGCTT	[29]
		R.Abbrev.COI.601r	AAAAAGAACAATTAAAATTACGATCC	

## Data Availability

The data supporting the conclusions of this article are included within the article.

## References

[1] Cox F.E.G. (2004). History of human parasitic diseases. Infect. Dis. Clin. N. Am..

[2] Cao B., Guiton P.S. (2018). Important Human Parasites of the Tropics. Front. Young Minds.

[3] Devaux C.A., Mediannikov O., Medkour H., Raoult D. (2019). Infectious Disease Risk Across the Growing Human-Non Human Primate Interface: A Review of the Evidence. Front. Public Health.

[4] Strait K., Else J.G., Eberhard M.L. (2012). Parasitic diseases of non human primates. Nonhuman Primates in Biomedical Research.

[5] Gómez J.M., Nunn C.L., Verdú M. (2013). Centrality in primate-parasite networks reveals the potential for the transmission of emerging infectious diseases to humans. Proc. Natl. Acad. Sci. USA.

[6] Ashford R.W., Reid G.D.F., Butynski T.M. (1990). The intestinal faunas of man and mountain gorillas in a shared habitat. Ann. Trop. Med. Parasitol..

[7] Muriuki S.M.K., Murugu R.K., Munene E., Karere G.M., Chai D.C. (1998). Some gastro-intestinal parasites of zoonotic (public health) importance commonly observed in old world non-human primates in Kenya. Acta Trop..

[8] Nizeyi J., Cranfield M., Graczyk T. (2002). Cattle near the Bwindi Impenetrable National Park, Uganda, as a reservoir of *Cryptosporidium parvum* and *Giardia duodenalis* for local community and free-ranging gorillas. Parasitol. Res..

[9] Leendertz F.H., Pauli G., Maetz-Rensing K., Boardman W., Nunn C., Ellerbrok H., Jensen S.A., Junglen S., Christophe B. (2006). Pathogens as drivers of population declines: The importance of systematic monitoring in great apes and other threatened mammals. Biol. Conserv..

[10] Mohammed Mahdy A.K., Lim Y.A.L., Surin J., Wan K.L., Al-Mekhlafi M.S.H. (2008). Risk factors for endemic giardiasis: Highlighting the possible association of contaminated water and food. Trans. R. Soc. Trop. Med. Hyg..

[11] Vlčková K., Kreisinger J., Pafčo B., Čížková D., Tagg N., Hehl A.B., Modrý D. (2018). Diversity of *Entamoeba* spp. in African great apes and humans: An insight from Illumina MiSeq high-throughput sequencing. Int. J. Parasitol..

[12] Hamad I., Forestier C.L., Peeters M., Delaporte E., Raoult D., Bittar F. (2015). Wild gorillas as a potential reservoir of *Leishmania major*. J. Infect. Dis..

[13] Medkour H., Davoust B., Levasseur A., Mediannikov O. (2019). Molecular Evidence of *Leishmania infantum* and *Leishmania guyanensis* in Red Howler Monkey (*Alouatta seniculus*) From French Guiana. Vector Borne Zoonotic Dis..

[14] Else J.G., Satzger M., Sturrock R.F. (1982). Natural infections of schistosoma mansoni and S. Haematobium in cercopithecus monkeys in kenya. Ann. Trop. Med. Parasitol..

[15] Faust C., Dobson A.P. (2015). Primate malarias: Diversity, distribution and insights for zoonotic *Plasmodium*. One Health.

[16] Liu W., Li Y., Learn G.H., Rudicell R.S., Robertson J.D., Keele B.F., Ndjango J.-B.N., Sanz C.M., Morgan D.B., Locatelli S. (2010). Origin of the human parasite *Plasmodium falciparum* in gorillas. Nature.

[17] Liu W., Li Y., Shaw K.S., Learn G.H., Plenderleith L.J., Malenke J.A., Sundararaman S.A., Ramirez M.A., Crystal P.A., Smith A.G. (2014). African origin of the malaria parasite *Plasmodium vivax*. Nat. Commun..

[18] Lambrecht F., Dunn F., Eyles D. (1961). Isolation of *Plasmodium knowlesi* from Philippine Macaques. Nature.

[19] Singh J., Ray A., Nair C. (1953). Isolation of a new strain of *Plasmodium knowlesi*. Nature.

[20] Chin W., Contacos P.G., Coatney G.R., Kimball H.R. (1965). A naturally acquired quotidian-type malaria in man transferable to monkeys. Science.

[21] Müller M., Schlagenhauf P. (2014). *Plasmodium knowlesi* in travellers, update 2014. Int. J. Infect. Dis..

[22] Sleeman J.M., Meader L.L., Mudakikwa A.B., Foster J.W., Patton S. (2000). Gastrointestinal Parasites of Mountain Gorillas (*Gorilla Gorilla Beringei*) in the Parc National Des Volcans, Rwanda. J. Zoo Wildl. Med..

[23] Bhagwant S. (2004). Human *Bertiella studeri* (family *Anoplocephalidae*) infection of probable Southeast Asian origin in Mauritian children and an adult. Am. J. Trop. Med. Hyg..

[24] Estrada A., Garber P.A., Rylands A.B., Roos C., Fernandez-Duque E., Di Fiore A., Anne-Isola Nekaris K., Nijman V., Heymann E.W., Lambert J.E. (2017). Impending extinction crisis of the world’s primates: Why primates matter. Sci. Adv..

[25] Zommers Z., Macdonald D.W., Johnson P.J., Gillespie T.R. (2013). Impact of human activities on chimpanzee ground use and parasitism (*Pan troglodytes*). Conserv. Lett..

[26] Wenz-Mücke A., Sithithaworn P., Petney T.N., Taraschewski H. (2013). Human contact in fl uences the foraging behaviour and parasite community in long-tailed macaques. Parasitology.

[27] Kowalewski M.M., Gillespie T.R. (2018). Primatology, Biocultural Diversity and Sustainable Development in Tropical Forests.

[28] Conly J.M., Johnston B.L. (2008). The infectious diseases consequences of monkey business. Can. J. Infect. Dis. Med. Microbiol..

[29] Laidoudi Y., Medkour H., Latrofa M.S., Davoust B., Sokhna C., Barciela A., Hernandez-Aguilar R.A., Raoult D., Otranto D., Mediannikov O. (2020). Zoonotic *Abbreviata caucasica* in Wild Chimpanzees (*Pan troglodytes verus*) from Senegal. Pathogens.

[30] Fenollar F., Mediannikov O. (2018). Emerging infectious diseases in Africa in the 21st century. New Microbes New Infect..

[31] Sow D., Parola P., Sylla K., Ndiaye M., Delaunay P., Halfon P., Camiade S., Dieng T., Tine R.C.K., Faye B. (2017). Performance of real-time polymerase chain reaction assays for the detection of 20 gastrointestinal parasites in clinical samples from Senegal. Am. J. Trop. Med. Hyg..

[32] Landsoud-Soukate J., Tutin C.E.G., Fernandez M. (1995). Intestinal parasites of sympatric gorillas and chimpanzees in the Lope Reserve, Gabon. Ann. Trop. Med. Parasitol..

[33] Lilly A.A., Mehlman P.T., Doran D. (2002). Intestinal parasites in gorillas, chimpanzees, and humans at Mondika research site, dzanga-ndoki national park, Central African Republic. Int. J. Primatol..

[34] Renquist D.M., Whitney R.A. (1987). Zoonoses acquired from pet primates. Vet. Clin. N. Am. Small Anim. Pract..

[35] Nygård K., Schimmer B., Søbstad Ø., Walde A., Tveit I., Langeland N., Hausken T., Aavitsland P. (2006). A large community outbreak of waterborne giardiasis—delayed detection in a non-endemic urban area. BMC Public Health.

[36] Gillespie T.R., Greiner E.C., Chapman C.A. (2004). Gastrointestinal Parasites of the Guenons of Western Uganda. J. Parasitol..

[37] Nolan M.J., Unger M., Yeap Y.T., Rogers E., Millet I., Harman K., Fox M., Kalema-Zikusoka G., Blake D.P. (2017). Molecular characterisation of protist parasites in human-habituated mountain gorillas (*Gorilla beringei beringei*), humans and livestock, from Bwindi impenetrable National Park, Uganda. Parasites Vectors.

[38] Drakulovski P., Bertout S., Locatelli S., Butel C., Pion S., Mpoudi-Ngole E., Delaporte E., Peeters M., Mallié M. (2014). Assessment of gastrointestinal parasites in wild chimpanzees (*Pan troglodytes troglodytes*) in southeast Cameroon. Parasitol. Res..

[39] Baker D.G. (2008). Parasites of non-human primates. Flynn’s Parasites of Laboratory Animals.

[40] Ruch T.C. (1961). Diseases of Laboratory Primates. Ann. Surg..

[41] Karim M.R., Wang R., Yu F., Li T., Dong H., Li D., Zhang L., Li J., Jian F., Zhang S. (2015). Multi-locus analysis of *Giardia duodenalis* from nonhuman primates kept in zoos in China: Geographical segregation and host-adaptation of assemblage B isolates. Infect. Genet. Evol..

[42] Van Den Berghe L., Chardome M., Peel E. (1964). The Filarial Parasites of the Eastern Gorilla in the Congo. J. Helminthol..

[43] Fernando Caserta Tencatt L., Ribeiro de Britto M., Simone Pavanelli C. (2016). Revisionary study of the armored catfish *Corydoras paleatus* (Jenyns, 1842) (Siluriformes: Callichthyidae) over 180 years after its discovery by Darwin, with description of a new species. Neotrop. Ichthyol. J..

[44] Pommier de Santi V., Briolant S., Mahamat A., Ilcinkas C., Blanchet D., de Thoisy B., Reynaud Y., Hyvert G., Lou Marié J., Edouard S. (2018). Q fever epidemic in Cayenne, French Guiana, epidemiologically linked to three-toed sloth. Comp. Immunol. Microbiol. Infect. Dis..

[45] Thompson P. Overview of Rift Valley Fever. https://www.merckvetmanual.com/generalized-conditions/rift-valley-fever/overview-of-rift-valley-fever.

[46] Mohan S., Mohan H., Mohan S. (2017). Infectious and Parasitic Diseases. Essent. Pathol. Dent. Stud..

[47] Chalifoux L.V. (1993). Filariasis, New World Primates.

[48] Bain O., Mutafchiev Y., Junker K., Guerrero R., Martin C., Lefoulon E., Uni S. (2015). Review of the genus *Mansonella* Faust, 1929 sensu lato *(Nematoda: Onchocercidae*), with descriptions of a new subgenus and a new subspecies. Zootaxa.

[49] Mediannikov O., Ranque S. (2018). Mansonellosis, the most neglected human filariasis. New Microbes New Infect..

[50] Agerholm J.S. (2013). *Coxiella burnetii* associated reproductive disorders in domestic animals-a critical review. Acta Vet. Scand..

[51] Sandosham A.A. (1951). On Two Helminths from the Orang Utan, *Leipertrema rewelli* n.g., n.sp. and *Dirofilaria immitis* (Leidy, 1856). J. Helminthol..

[52] Rodhain J. (1947). Corollaire à l’étude de E. Peel et M. Chardome sur les filaridés des chimpanzés au Congo belge. Ann. Soc. Belge Med. Trop..

[53] Park N., Gillespie T.R., Lonsdorf E.V., Canfield E.P., Meyer D.J., Nadler Y., Raphael J., Pusey A.E., Pond J., Pauley J. (2014). Demographic and ecological effects on patterns of parasitism in eastern chimpanzees (*Pan troglodytes schweinfurthii*) in Gombe National Park, Tanzania. Am. J. Phys. Anthropol..

[54] Muehlenbein M.P. (2005). Parasitological analyses of the male chimpanzees (*Pan troglodytes schweinfurthii*) at Ngogo, Kibale National Park, Uganda. Am. J. Primatol..

[55] Petrželkov K.J., Hasegawa H., Appleton C.C., Huffman M.A., Archer C.E., Moscovice L.R., Mapua M.I., Singh J., Kaur T. (2010). Gastrointestinal parasites of the chimpanzee population introduced onto Rubondo Island National Park, Tanzania. Am. J. Primatol..

[56] Hasegawa H., Sato H., Fujita S., Nguema P.P.M., Nobusue K., Miyagi K., Kooriyama T., Takenoshita Y., Noda S., Sato A. (2010). Molecular identification of the causative agent of human strongyloidiasis acquired in Tanzania: Dispersal and diversity of *Strongyloides* spp. and their hosts. Parasitol. Int..

[57] Thanchomnang T., Intapan P.M., Sanpool O., Rodpai R., Tourtip S., Yahom S., Kullawat J., Radomyos P., Thammasiri C., Maleewong W. (2017). First molecular identification and genetic diversity of *Strongyloides stercoralis* and *Strongyloides fuelleborni* in human communities having contact with long-tailed macaques in Thailand. Parasitol. Res..

[58] Hamad I., Keita M.B., Peeters M., Delaporte E., Raoult D., Bittar F. (2014). Pathogenic eukaryotes in gut microbiota of western lowland gorillas as revealed by molecular survey. Sci. Rep..

[59] Hasegawa H., Modrý D., Kitagawa M., Shutt K.A., Todd A., Kalousová B., Profousová I., Petrželková K.J. (2014). Humans and Great Apes Cohabiting the Forest Ecosystem in Central African Republic Harbour the Same Hookworms. PLoS Negl. Trop. Dis..

[60] Levine N.D. (1968). Diseases of Man Acquired from His Pets. Am. J. Trop. Med. Hyg..

[61] Yamaguti S. (1962). Systema Helminthum. The Nematodes of Vertebrates.

[62] Rodrigo R.K., Perera M.S.J., Perera N., Peries T.N., de Silva S., Bambaradeniya C.N.B. (2008). A preliminary survey on the herpetofauna in the Anawilundawa wetland sanctuary: The sond Ramsar site of Sri Lanka. Tigerpaper.

[63] Essa A. (2004). Worms and human disease. J. Clin. Pathol..

[64] Vyas R. (2002). Preliminary survey of herpetofauna of Narayan sarovar Sanctuary, Gujarat. Zoos’ Print J..

[65] Remfry J. (1978). The incidence, pathogenesis and treatment of helminth infections in rhesus monkeys (*Macaca mulatta*). Lab. Anim..

[66] Toft J.D., Benirschke K. (1986). The pathoparasitology of nonhuman primates: A review. Primates.

[67] Yamashita J. (1963). Ecological relationships between parasites and primates—I. Helminth Parasites and Primates. Primates.

[68] Polderman A.M., Blotkamp J. (1995). *Oesophagostomum* infections in humans. Parasitol. Today.

[69] Ghai R.R., Chapman C.A., Omeja P.A., Davies T.J., Goldberg T.L. (2014). Nodule Worm Infection in Humans and Wild Primates in Uganda: Cryptic Species in a Newly Identified Region of Human Transmission. PLoS Negl. Trop. Dis..

[70] Dridi B., Henry M., El Khéchine A., Raoult D., Drancourt M. (2009). High prevalence of *Methanobrevibacter smithii* and *Methanosphaera stadtmanae* detected in the human gut using an improved DNA detection protocol. PLoS ONE.

[71] Medkour H., Varloud M., Davoust B., Mediannikov O. (2020). New Molecular Approach for the Detection of Kinetoplastida Parasites of Medical and Veterinary Interest. Microorganisms.

[72] Medkour H., Laidoudi Y., Athias E., Bouam A., Dizoé S., Davoust B., Mediannikov O. (2020). Molecular and serological detection of animal and human vector-borne pathogens in the blood of dogs from Côte d’Ivoire. Comp. Immunol. Microbiol. Infect. Dis..

[73] Dahmana H., Amanzougaghene N., Davoust B., Normand T., Carette O., Demoncheaux J.P., Mulot B., Fabrizy B., Scandola P., Chik M. (2019). Great diversity of Piroplasmida in Equidae in Africa and Europe, including potential new species. Vet. Parasitol. Reg. Stud. Rep..

[74] Verweij J.J., Laeijendecker D., Brienen E.A.T., Van Lieshout L., Polderman A.M. (2003). Detection of *Cyclospora cayetanensis* in travellers returning from the tropics and subtropics using microscopy and real-time PCR. Int. J. Med. Microbiol..

[75] Mourembou G., Fenollar F., Socolovschi C., Lemamy G.J., Nzoughe H., Kouna L.C., Toure-Ndouo F., Million M., Mbiguino A.N., Lekana-Douki J.B. (2015). Molecular detection of fastidious and common Bacteria as well as *Plasmodium* spp. in Febrile and Afebrile children in Franceville, Gabon. Am. J. Trop. Med. Hyg..

[76] Jauregui L.H., Higgins J., Zarlenga D., Dubey J.P., Lunney J.K. (2001). Development of a real-time PCR assay for detection of *Toxoplasma gondii* in pig and mouse tissues. J. Clin. Microbiol..

[77] Garcés-Sanchez G., Wilderer P.A., Munch J.C., Horn H., Lebuhn M. (2009). Evaluation of two methods for quantification of hsp70 mRNA from the waterborne pathogen *Cryptosporidium parvum* by reverse transcription real-time PCR in environmental samples. Water Res..

[78] Verweij J.J., Blange R.A., Templeton K., Schinkel J., Brienen E.A.T., Van Rooyen M.A.A., Van Lieshout L., Polderman A.M. (2004). *Cryptosporidium parvum* in fecal samples by using Multiplex real-time PCR. J. Clin. Microbiol..

[79] Roy S., Kabir M., Mondal D., Ali I.K.M., Petri W.A., Haque R. (2005). Real-time-PCR assay for diagnosis of *Entamoeba histolytica* infection. J. Clin. Microbiol..

[80] Laidoudi Y., Davoust B., Varloud M., Niang E.H.A., Fenollar F., Mediannikov O. (2020). Development of a multiplexed qPCRs-based approach for the diagnosis of *Dirofilaria immitis, D. repens, Acanthocheilonema reconditum* and the others filariosis. bioRxiv.

[81] Bassene H., Sambou M., Fenollar F., Clarke S., Djiba S., Mourembou G., Alioune Badara L.Y., Raoult D., Mediannikov O. (2015). High prevalence of *Mansonella perstans* filariasis in rural Senegal. Am. J. Trop. Med. Hyg..

[82] Touré F.S., Bain O., Nerrienet E., Millet P., Wahl G., Toure Y., Doumbo O., Nicolas L., Georges A.J., McReynolds L.A. (1997). Detection of *Loa loa*-specific DNA in blood from occult-infected individuals. Exp. Parasitol..

[83] Verweij J.J., Brienen E.A.T., Ziem J., Yelifari L., Polderman A.M., Van Lieshout L. (2007). Simultaneous detection and quantification of *Ancylostoma duodenale, Necator americanus*, and *Oesophagostomum bifurcum* in fecal samples using multiplex real-time PCR. Am. J. Trop. Med. Hyg..

[84] Wichmann D., Panning M., Quack T., Kramme S., Burchard G.D., Grevelding C., Drosten C. (2009). Diagnosing schistosomiasis by detection of cell-free parasite DNA in human plasma. PLoS Negl. Trop. Dis..

[85] Praet N., Verweij J.J., Mwape K.E., Phiri I.K., Muma J.B., Zulu G., van Lieshout L., Rodriguez-Hidalgo R., Benitez-Ortiz W., Dorny P. (2013). Bayesian modelling to estimate the test characteristics of coprology, coproantigen ELISA and a novel real-time PCR for the diagnosis of taeniasis. Trop. Med. Int. Health.

[86] Wiria A., Indonesia J., Leiden T., Prasetyani M., Hamid F., University M., Wammes L., Lell B., Hospital A., Ariawan I. (2010). Does treatment of intestinal helminth infections influence malaria? Background and methodology of a longitudinal study of clinical, parasitological and immunological parameters in Nangapanda, Flores, Indonesia (ImmunoSPIN Study). BMC Infect. Dis..

[87] Liu J., Gratz J., Amour C., Kibiki G., Becker S., Janaki L., Verweij J.J., Taniuchi M., Sobuz S.U., Haque R. (2013). A laboratory-developed taqman array card for simultaneous detection of 19 enteropathogens. J. Clin. Microbiol..

[88] Verweij J.J., Canales M., Polman K., Ziem J., Brienen E.A.T., Polderman A.M., van Lieshout L. (2009). Molecular diagnosis of *Strongyloides stercoralis* in faecal samples using real-time PCR. Trans. R. Soc. Trop. Med. Hyg..

[89] Laidoudi Y., Ringot D., Watier-Grillot S., Davoust B., Mediannikov O. (2019). A cardiac and subcutaneous canine dirofilariosis outbreak in a kennel in central France. Parasite.

